# Medical Student Balint – a Tool to Improve Empathy, Transform Communication Skills and Empower Tomorrow’s Socially Aware Patients’ Advocates

**DOI:** 10.1192/bjo.2025.10321

**Published:** 2025-06-20

**Authors:** Justyna Wroblewska, Burak Cardak, Michael Milmore

**Affiliations:** RDASH, Sheffield, United Kingdom

## Abstract

**Aims:** A trial of Balint Group for eleven medical students during their psychiatry placement in Rotherham, UK, to add to the existing pool of evidence regarding the impact of Balint Group on empathy, communication skills, understanding of doctor-patient interaction and to improve the understanding of the wider socio-political context of patients, with the hope of developing agency, consultation abilities, advocacy and well-being among future doctors.

**Methods:** Balint Groups were facilitated by two Psychiatry Core Trainees with weekly supervision by a consultant medical psychotherapist. Five sessions were integrated into the students’ weekly timetable. The students were sent questionnaires before and after the pilot, asking for views on the role of psychological factors in the doctor-patient interaction as well as Balint Groups. There were seven closed questions in both questionnaires and an extra two open questions in the ending questionnaire.

**Results:** Three students attended all sessions, with others having inconsistent attendance, resulting in six to ten students in each session. With each subsequent session, the students displayed more openness and reflection during discussions. Ten students answered the initial questionnaire and nine the ending questionnaire. Therapeutic relationships were discussed in the context of wider issues, such as abortion, homophobia, migration and racism, menopause, and prejudice from healthcare professionals towards patients from lower socio-economic backgrounds. In answers to the open questions, the students highlighted the beneficial effects of the sessions on their subjective levels of empathy, better understanding of psychosocial factors involved in patients’ presentation, benefits of reflection to help resolve internal conflicts, reduce burnout and create solidarity between students. However, there was no significant change in the answers to the closed questions. This could be a result of inconsistent attendance, short duration of trial and ambiguity of questions. The students were, on average, already highly aware of the role psychological factors play in patients’ presentation and had some awareness of the role of doctors’ attitudes towards patients, which could have contributed to this also. Both trainees found their own benefits facilitating the sessions; improving their leadership, organisation and psychotherapy skills.

**Conclusion:** This five-week Balint Group pilot produced benefits described by the medical students as improved empathy, psychosocial awareness, reflective practice, reducing burnout and improving peer to peer bonds. The pilot also demonstrated the role of Balint Groups as an educational tool to enhance sociopolitical understanding; empowering doctors as advocates and addressing harmful biases that can otherwise persist in healthcare settings.

